# Analysis of cross-provincial healthcare utilization among hospitalized patients in a tertiary Chinese medicine hospital

**DOI:** 10.3389/fpubh.2026.1818250

**Published:** 2026-08-17

**Authors:** Luqing Zeng, Junying Wang, Hua Zhang, Jian Liang, Hanhua Bai, Yanbin Yang

**Affiliations:** 1 Medical Records and Statistics Department, The Eighth Clinical Medical College of Guangzhou University of Chinese Medicine, Foshan Hospital of Traditional Chinese Medicine, Foshan, Guangdong, China; 2 Social Health Insurance Department, The Eighth Clinical Medical College of Guangzhou University of Chinese Medicine, Foshan Hospital of Traditional Chinese Medicine, Foshan, Guangdong, China; 3 Department of Medical Quality Management, Foshan First People's Hospital, Foshan, Guangdong, China; 4 Department of Science and Education, The Eighth Clinical Medical College of Guangzhou University of Chinese Medicine, Foshan Hospital of Traditional Chinese Medicine, Foshan, Guangdong, China

**Keywords:** circular distribution, cross-provincial healthcare utilization, disease spectrum, grey relational analysis, hospitalized patients, spatial distribution

## Abstract

**Objective:**

To analyze the current situation and changing trends of cross-provincial healthcare utilization among hospitalized patients in a tertiary Chinese medicine hospital, explore the supply and demand characteristics of these patients, and provide references for improving the management of cross-provincial medical services and the refined management of the hospital.

**Methods:**

Based on data extracted from the Guangdong Provincial Medical Record Information System, we collected medical records of hospitalized patients with cross-provincial healthcare utilization from a tertiary Chinese medicine hospital between 2016 and 2025. We analyzed the characteristics of these patients, including the number of visits, spatial distribution of origin, departmental distribution, spectrum of diseases, and cost implications.

**Results:**

From 2016 to 2025, the total number of inpatient visits for cross-provincial medical treatment was 67,835. The majority of patients were male, accounting for 62.94%. The most affected age group was the young and middle-aged population (31–60 years). Regarding medical insurance types, self-pay (out-of-pocket) patients were the most common, accounting for 55.60% of the total, though this proportion showed a decreasing trend year by year. The length of hospital stay was predominantly 0–10 days, accounting for 70.72% of all cases. The peak period for hospital discharge was August 17. The primary source province was Hunan Province, accounting for 21.51%. Spatial autocorrelation analysis showed that the global Moran’s I index was consistently greater than 0, indicating positive spatial autocorrelation and spatial clustering. Spatial hotspot analysis identified Guizhou, Chongqing, and Hunan as hotspot regions. The main inpatient department was orthopedics and traumatology, accounting for 49.25% of all admissions. In terms of disease classification, the most common category was injuries, poisoning, and certain other consequences of external causes (22,298 person-times). The average hospitalization cost per visit showed little difference between cross-provincial patients and local patients. Grey relational analysis indicated that consumables cost had the greatest influence on the average hospitalization cost per visit, and the proportion of consumables cost showed a decreasing trend year by year.

**Conclusion:**

The demand for cross-provincial inpatient care is continuously increasing. Hospitals should integrate this trend into their medical management strategies and new service development. It is essential to optimize discipline allocation, enhance core competitiveness, expand the hospital’s reach and influence, and increase publicity regarding cross-provincial healthcare policies to provide high-quality medical services to a broader national patient population.

## 1 Introduction

Cross-regional healthcare is an inevitable trend in social development, Many healthcare systems provide patients with cross-regional, cross-national, and transcontinental services. The European Union (EU) countries are the birthplace of modern social security systems. The Court of Justice of the European Union (CJEU) has established relevant policies to safeguard the rights of European residents to access healthcare services within EU member states (1–3). For a long time, medical resources in China have been unevenly distributed. With the rapid development of the national economy, increasing public demand for health, deepening reform processes, advancing urbanization, growing population mobility, and the realization of convenient transportation networks, the phenomenon of cross-provincial healthcare utilization has increased significantly (4–10). The direct settlement for cross-provincial healthcare is a major initiative proposed by the State Council and a crucial measure to address the challenges in medical expense settlement for insured individuals seeking cross-provincial care, which arise from the uneven distribution of medical resources and the localized management of healthcare insurance in China (11–13).

The COVID-19 pandemic has posed an unprecedented stress test to global healthcare systems, prompting the academic community to reassess the connotation and implementation pathways of healthcare system resilience. Evidence from Europe indicates that during the pandemic, fiscal resources were sharply redirected toward epidemic response, profoundly revealing the tension between emergency budget allocation and the long-term sustainability of healthcare systems, while relying solely on emergency fiscal measures proved inadequate to address systemic challenges (14). The Romanian case further corroborates this, showing that the financing system experienced budgetary imbalances under the impact of the pandemic, and routine management optimization measures proved highly vulnerable, necessitating a systematic reconstruction of system resilience from the dimensions of governance effectiveness, resource allocation adaptability, and crisis learning capacity (15). These insights provide a comparative perspective for analyzing the phenomenon of cross-provincial healthcare utilization in China: although mobility restrictions during the pandemic placed cross-provincial “medical migrants” under significant pressure, national data indicate that the long-term growth trend of cross-provincial inpatient demand has not been reversed. Moreover, cross-provincial patients exhibit higher demand elasticity compared with local patients, making them more susceptible to displacement by external disruptions. Therefore, this paper integrates “healthcare system resilience” and “healthcare demand elasticity” into a unified theoretical analytical framework—the former examining the system’s ability to maintain core functions and undergo adaptive reorganization under shocks, and the latter characterizing the differentiated responses of various patient groups to external disruptions. This framework aims to provide a theoretical basis for balancing the strategy of regional medical centers in safeguarding local essential demand while maintaining cross-provincial attractiveness in the post-pandemic era.

Guangdong Province has a developed economy overall, particularly in the Pearl River Delta region, where high-quality medical resources are highly concentrated, hosting nationally renowned medical institutions in South China and even nationwide. Foshan is a nationally renowned historical and cultural city and a key manufacturing hub in the Guangdong-Hong Kong-Macao Greater Bay Area, with a substantial cross-provincial floating population. It is one of the primary destinations for population inflow in the Pearl River Delta, with Guangxi being the largest source region, Hunan also being a significant source, and considerable populations from Sichuan, Guizhou, Jiangxi, Henan, and other provinces (16). As a vital traditional Chinese medicine (TCM) medical center in the Guangdong-Hong Kong-Macao Greater Bay Area, Foshan Hospital of Traditional Chinese Medicine has, in recent years, been the TCM institution in the province that has admitted the highest number of discharge cases among critical and complicated cross-provincial patients. The hospital is characterized by its strong TCM features and has multiple national and provincial key specialties, including four national-level key specialties: Orthopedics and Traumatology, Endocrinology, Encephalopathy (Neurology), and Oncology. Its musculoskeletal specialty, trauma specialty, and polytrauma specialty all rank among the top three in the province. Convenient transportation and high-quality medical resources endow the hospital with a strong capacity to attract patients from surrounding provinces. Therefore, it is essential to conduct relevant research to provide better quality medical services for cross-provincial patients.

This study analyzed the data of cross-provincial inpatients discharged from the hospital between January 1, 2016, and December 31, 2025. The analysis includes: (1) a comprehensive analysis of the total volume of cross-provincial patients, including trends in inpatient visits and hospitalization costs; (2) an analysis of the demographic characteristics of this patient population, including gender, age, marital status, type of medical insurance, and trends and proportions of length of hospital stay; (3) a circular distribution analysis of discharge dates; (4) a spatial analysis of source provinces, including distribution characteristics, spatial autocorrelation analysis, and hotspot analysis; (5) an analysis of inpatient departments visited; (6) a disease spectrum analysis, including systemic diseases and the top 20 disease categories; and (7) a cost analysis, including comparisons of per capita costs between local and cross-provincial patients, trends and proportions of various cost items, and grey relational analysis examining the degree of association between per capita hospitalization costs and individual cost components.

The contributions of this paper are mainly reflected in the following aspects. First, in terms of research perspective, existing literature has predominantly analyzed cross-regional healthcare utilization from the perspective of Western general hospitals or at the overall level of non-local medical care, with limited dedicated attention to the utilization characteristics of cross-provincial inpatients in traditional Chinese medicine (TCM) hospitals as a specific type of institution. TCM hospitals have unique characteristics in service models, patient composition, and medical resource distribution, and the spatial patterns and driving mechanisms of cross-provincial healthcare utilization in TCM hospitals may differ significantly from those in general hospitals. Second, in terms of theoretical framework, this study introduces healthcare system resilience analysis into cross-provincial healthcare utilization research. By comparing the differentiated responses of cross-provincial and local patient demand during the pandemic, it reveals that the “demand elasticity” of cross-provincial healthcare utilization can serve as a sensitive indicator for measuring changes in the attractiveness of regional TCM medical centers, providing theoretical reference for assessing the functional positioning and resource allocation strategies of TCM hospitals in the post-pandemic era. Third, at the empirical level, this study is based on inpatient data from Foshan Hospital of Traditional Chinese Medicine. As a regional medical center that has ranked first among prefecture-level TCM hospitals nationwide for 12 consecutive years, the spatiotemporal evolution characteristics of its cross-provincial healthcare utilization are representative for understanding the efficiency of cross-regional allocation of TCM medical resources. The findings can provide empirical evidence for optimizing non-local medical service management and medical insurance policies for TCM hospitals.

## 2 Materials and methods

### 2.1 Data sources

The data were sourced from the medical record front page information of cross-provincial healthcare inpatients discharged between January 1, 2016, and December 31, 2025, within the medical record information system of a tertiary Chinese medicine hospital. A total of 67,835 cases with both “current address” and “registered domicile” outside Guangdong Province were included. Cases with missing, duplicate, or logically inconsistent data were excluded (0 cases), ultimately yielding 67,835 valid cases. Patient demographic characteristics such as age, gender, marital status, type of medical insurance, and place of origin were collected, along with hospitalization service features including length of hospital stay, department of admission, discharge diagnosis, and hospitalization expenses. Meanwhile, the medical record homepage information of local hospitalized patients discharged between January 1, 2016 and December 31, 2025 was also collected. Cases in which both “current address” and “registered residence address” were “Foshan City, Guangdong Province” were included. Cases with missing data, duplicates, or logical inconsistencies were excluded, resulting in a final sample of 294,430 valid cases. The average cost per case for these patients was collected. The specific rules for data extraction are as follows: (1) Duplicate inpatient cases: For each patient hospitalization episode, only the first admission record was retained. Duplicate records (e.g., multiple entries for the same hospitalization event) were excluded. (2) Definition of emergency/acute care: Both emergency admissions and elective admissions were included in this study to comprehensively capture cross-provincial inpatient utilization. No exclusions were made based on admission type. (3) Handling of missing values: Cases with missing key variables (e.g., age, sex, discharge diagnosis, hospitalization cost) were excluded using listwise deletion. The proportion of excluded cases accounted for less than 2% of the total sample. (4) Definition of “cross-provincial inpatients”: A cross-provincial inpatient was defined as a patient whose registered domicile (hukou address) and current residential address were both not in Guangdong Province. This dual-address criterion was adopted to ensure the inclusion of patients who truly crossed provincial boundaries for medical care, while avoiding reliance on medical insurance registration location (which may not reflect actual place of residence). We have acknowledged in the Limitations section that this definition may exclude the migrant population with out-of-province registered domicile but long-term residence in Guangdong.

### 2.2 Statistical methods

#### 2.2.1 Descriptive analysis

Descriptive analyses were performed using Microsoft Excel. These included analyses of the sex composition, age distribution, departmental distribution, and disease spectrum among cross-provincial healthcare inpatients. Additionally, annual trends in the number of hospitalizations, total hospitalization costs, and the composition of these costs were examined. The SPSS software was used to analyze the differences in average cost per hospitalization between cross-provincial hospitalized patients and local hospitalized patients.

#### 2.2.2 Circular distribution analysis

Circular distribution analysis is the optimal method for analyzing the temporal distribution characteristics of discharge dates among cross-provincial inpatients, for three main reasons. First, discharge dates constitute periodic directional data (January 1 and December 31 are connected end-to-end in terms of seasonal patterns), and circular distribution analysis is specifically designed for such cyclic structures, enabling accurate identification of central tendency. Second, by calculating the mean angle and degree of dispersion (r-value), this method can precisely locate peak discharge periods, providing a clear basis for hospital resource allocation in areas such as bed turnover, settlement window scheduling, and medical insurance review. Third, this method has been widely applied in hospital administration, supported by Rayleigh’s uniformity test and Watson-Williams test for comparing concentrations across different years, ensuring the statistical reliability of analytical conclusions and comparability across years. Circular distribution analysis (17) was implemented using Microsoft Excel to examine the temporal discharge patterns of cross-provincial healthcare patients. Periodic monthly data were converted into linear data via trigonometric functions. The mean angle was calculated to measure central tendency, and the Rayleigh’s Z value served as the test statistic (18, 19). The analysis using the circular distribution method follows the steps outlined below: (1) Convert the 12 months of a year into 360°, where one month and one day correspond to 30° and 0.9863°, respectively. The class midpoint is represented by the middle value of each month and converted into the corresponding degree. The average converted angles for each month are 15°, 45°, 75°, and so on. Alternatively, convert the 24 h of a day into 360°, where one hour and one minute correspond to 15° and 0.25°, respectively. The class midpoint is represented by the middle value of each hour and converted into the corresponding degree. The average converted angle *α* for each hour is 7.7°, 22.5°, 37.5°, and so forth. (2) Calculate the mean values of cos α and sin α, denoted as X and Y, respectively, to obtain the mean angle α. Compute the standard deviation S of the angle, which represents the dispersion trend among the angles in the circular distribution, and then calculate the 95% confidence interval for the mean angle. (3) Perform hypothesis testing on the significance level of the mean angle using Rayleigh’s test, with a significance level of *α* = 0.05. When the data do not meet the applicability conditions of the traditional circular distribution analysis method (i.e., unimodal or approximately unimodal distribution), a modified circular distribution analysis method is used to analyze the data. The specific statistical calculation methods are as follows:

X=(∑fcosa)/n

Y=(∑fcosa)/n

$$\gamma =\sqrt{X^{2}+Y^{2}}$$

sin.¯ɑ = Y/*γ*,cos.ɑ = X/γ

$$S=180^{{}^{\circ}}/\pi \sqrt{-2\mathrm{ln\gamma}}\;\mathrm{或S}=122.9548^{{}^{\circ}}\sqrt{-\mathrm{Lg\gamma}}$$

$$Z=n\gamma^{2},Z>Z_{0.05}$$

#### 2.2.3 Spatial distribution analysis

Geoda 1.22.0.10 was employed to conduct spatial distribution analysis of patients’ source provinces. First, a distribution map of source provinces of relevant cases was established, using colors of varying shades to represent the volume of cross-provincial inpatient visits, thereby reflecting high-incidence and low-incidence areas. Second, spatial autocorrelation analysis (20) was applied for quantitative assessment. The Global Moran’s I was used to diagnose whether the spatial association pattern of cross-provincial inpatient visits was clustered, dispersed, or randomly distributed, as well as the strength and significance of this association (21). The Global Moran’s I ranges from −1 to 1. When the index is less than 0, it indicates a negative correlation, with values closer to −1 signifying greater differences among units and a more dispersed distribution. When the index equals 0, it indicates no correlation among units. When the index is greater than 0, it indicates a positive correlation, with values closer to 1 signifying smaller differences among units and a more clustered distribution. The significance level of the Moran’s I index was tested using the standardized Z(I) statistic, with a significance level of *α* = 0.05. Finally, spatial hotspot analysis was employed to further identify hotspot and coldspot areas of cross-provincial inpatient visits. The Getis-Ord Gi* statistic was calculated, and based on the sign and statistical significance of this metric, the presence of statistically significant high-value and low-value clusters was assessed (22). When calculating the Moran’s I and Getis-Ord Gi* statistics, the spatial weights matrix was specified as a contiguity matrix. Moran’s I is well-suited for assessing the geographic concentration of patients, for the following main reasons. First, this metric effectively measures the spatial autocorrelation of patient source regions—that is, it determines whether areas with high patient volumes are geographically adjacent to each other and form clusters, rather than being randomly distributed. Second, the Global Moran’s I can test whether overall spatial clustering is statistically significant, while the Local Moran’s I (LISA) can precisely pinpoint the core high-value clusters that attract patients. Third, this method naturally aligns with the distance decay principle of cross-provincial healthcare utilization, as it can quantify the impact of geographic proximity on the distribution of patient volumes through spatial weights matrices, and has been well-established in spatial analysis applications within the public health field.

#### 2.2.4 Grey relational analysis

The advantages of grey relational analysis in the cost structure analysis of per capita hospitalization expenses for cross-provincial patients are mainly reflected in three aspects. First, it does not require independence among cost indicators, effectively avoiding the distortion of regression coefficients caused by multicollinearity, and allows for the direct inclusion of all cost items—such as medication costs, examination costs, and material costs—into the analysis simultaneously. Second, it does not rely on large sample sizes or the assumption of normal distribution. Instead, by comparing the geometric curve similarity between each cost sub-sequence and the total cost sequence, it ranks the key cost items that influence per capita hospitalization expenses by their degree of association. Third, the “relational order” it produces directly indicates the priority directions for cost control, offering greater management decision-making relevance than marginal effect analysis. This approach is particularly well-suited for exploratory research with complex cost structures and limited sample sizes. Grey relational analysis was conducted using SPSS to evaluate the associations between the average cost per hospitalization for cross-provincial patients and its constituent cost items (23, 24). As a key method for quantifying the strength of relationships between factors, it provides insights into the relative closeness of various influencing factors to the average hospitalization cost. A higher relational degree (r) corresponds to a greater degree of influence. The analysis steps are as follows: (1) Determine the parent sequence and subsequences; (2) To reduce the influence of dimensionality on the results and to simplify calculations, data preprocessing is required. Preprocessing methods include positive transformation, standardization, normalization, etc.; (3) Calculate the grey correlation coefficients; (4) Calculate the average of the correlation coefficients, and finally rank the degrees of correlation. In this study, the resolution coefficient is set to 0.5.

## 3 Results

### 3.1 Volume and trends of cross-provincial healthcare patients

**Figure 1:**
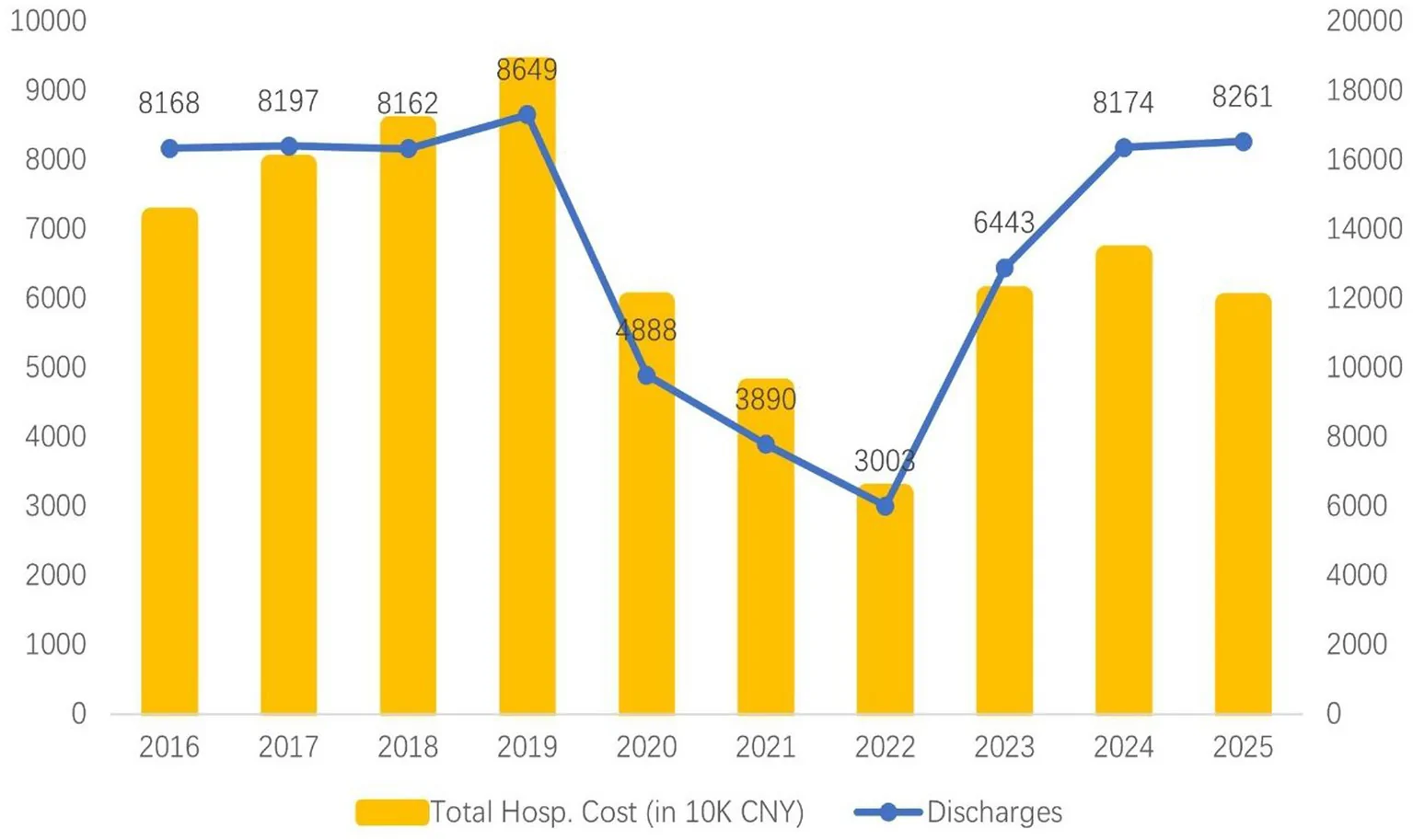
Hospitalization service profile of cross-provincial healthcare patients (2016–2025).

Analysis of hospitalization services for cross-provincial patients revealed that, influenced by the COVID-19 pandemic, the annual number of such visits to the hospital remained below 8,000 from 2020 to 2023. In contrast, the number exceeded 8,000 in all other years of the study period. Concurrently, total hospitalization costs demonstrated an overall declining trend (Figure 1).

### 3.2 Basic characteristics of cross-provincial healthcare patients

Analysis of the demographic characteristics of cross-provincial inpatients revealed several key patterns. Annually, the proportion of male patients exceeded that of females. Cumulatively from 2016 to 2025, males accounted for 62.94% of patients, compared to 37.06% for females. The age group 46–60 years constituted the largest proportion (37.01%), followed by the 31–45 age group (31.33%). Married was the predominant marital status, representing 81.81% of patients. Regarding payment, self-pay (out-of-pocket) was the primary insurance type, comprising 55.60% of cases. The majority of hospital stays (70.72%) lasted between 0 and 10 days (Table 1). The surge in the “Other” category of insurance type from 2023 to 2025 is not a true epidemiological change, but an artifact caused by adjustments to the hospital’s EMR system fields.

**Table 1:** Basic characteristics of cross-provincial inpatients (2016–2025).

Item	Discharges											PCT/%
	2016	2017	2018	2019	2020	2021	2022	2023	2024	2025	Total	
Sex												
Male	5,270	5,318	5,271	5,440	3,137	2,552	1932	3,884	4,856	5,036	42,696	62.94
Female	2,898	2,879	2,891	3,209	1751	1,338	1,071	2,559	3,318	3,225	25,139	37.06
Age												
0–18 years	519	446	394	475	214	161	105	313	420	368	3,415	5.03
19–30 years	1,505	1,341	1,138	1,146	533	421	261	630	780	710	8,465	12.48
31–45 years	3,103	2,930	2,874	2,711	1,565	1,169	886	1722	2,143	2,150	21,253	31.33
46–60 years	2,262	2,627	2,824	3,165	1934	1,581	1,292	2,602	3,380	3,441	25,108	37.01
60 + years	779	853	932	1,152	642	558	459	1,176	1,451	1,592	9,594	14.14
Marital status												
Married	6,935	7,083	6,576	6,579	3,759	3,055	2,402	5,300	6,829	6,977	55,495	81.81
Single	1,184	1,044	1,049	1,163	594	502	308	900	1,200	1,142	9,086	13.39
Other	2	7	426	796	432	261	223	172	81	72	2,472	3.64
Divorced	22	37	67	51	49	34	36	36	32	40	404	0.60
Widowed	25	26	44	60	54	38	34	35	32	30	378	0.56
Insurance type												
Social basic medical insurance	1,054	1,073	1,432	2094	1,514	1,361	1,375	4,404	4,374	3,820	22,501	33.17
Commercial health insurance	0	0	0	0	6	59	47	1	2	0	115	0.17
Out-of-pocket	7,112	7,124	6,730	6,552	3,327	2,442	1,476	1,050	1,070	830	37,713	55.60
Other	2	0	0	3	41	28	105	988	2,728	3,611	7,506	11.07
Length of hospital stay												
0–10 days	5,387	5,327	5,404	5,979	3,453	2,772	2,127	4,883	6,255	6,387	47,974	70.72
11–20 days	1971	1968	1990	2005	1,069	847	655	1,217	1,543	1,522	14,787	21.80
21–30 days	450	522	501	449	232	170	147	227	246	243	3,187	4.70
31–40 days	184	178	128	103	72	49	32	64	73	61	944	1.39
40 + days	176	202	139	113	62	52	42	52	57	48	943	1.39

### 3.3 Distribution of discharge times for cross-provincial inpatients

**Figure 2:**
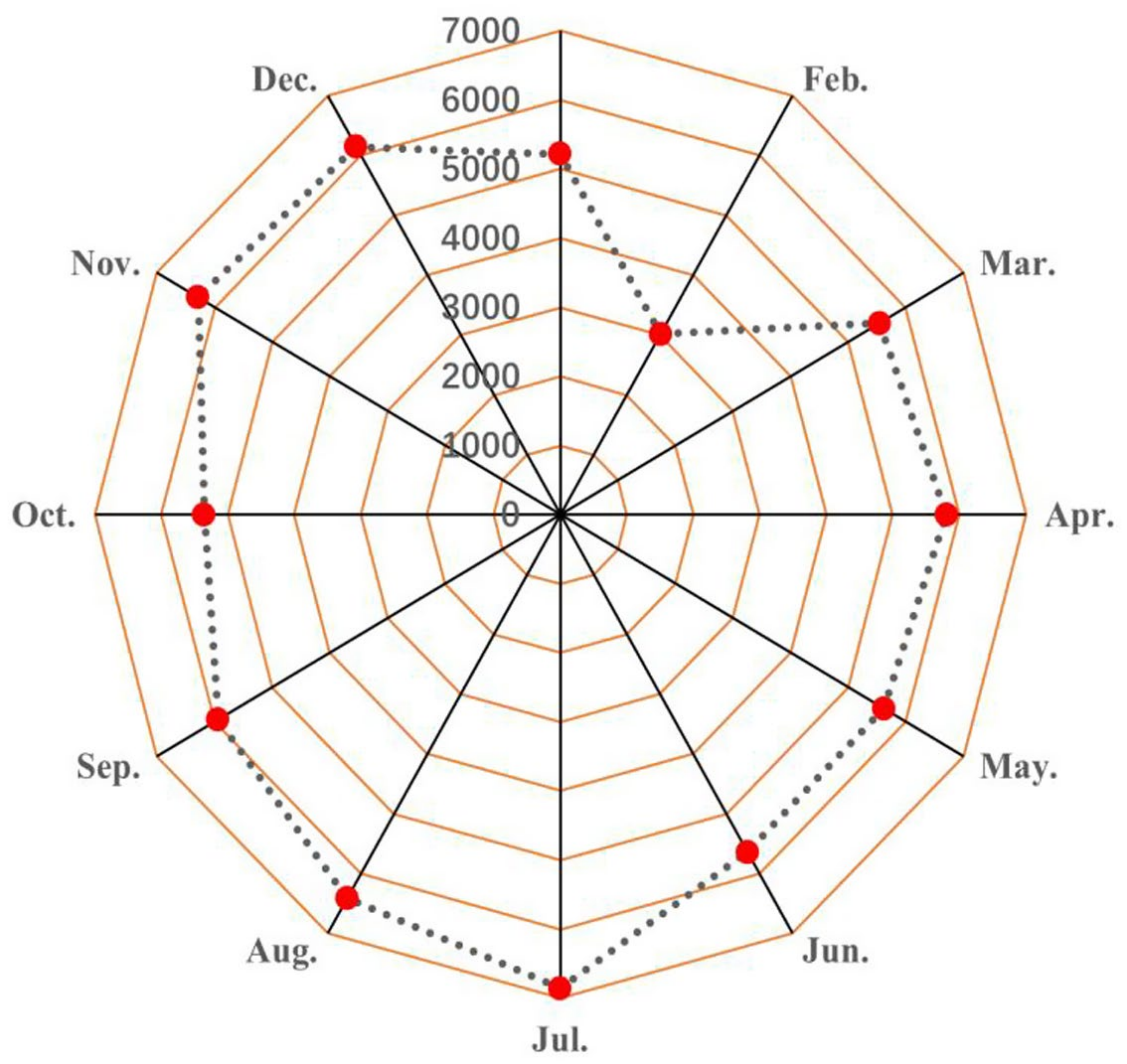
Circular distribution of discharge months for cross-provincial healthcare patients (2016–2025).

The concentration parameter (r) for the monthly distribution of cross-provincial patient discharges from 2016 to 2025 was 0.0688. The Rayleigh’s test indicated a statistically significant departure from a uniform circular distribution (Z = 320.7902, *p* < 0.05), confirming the presence of a mean direction. The peak discharge date was calculated to be August 17 (Figure 2).

### 3.4 Spatial distribution of provinces of origin for cross-provincial inpatients

**Figure 3:**
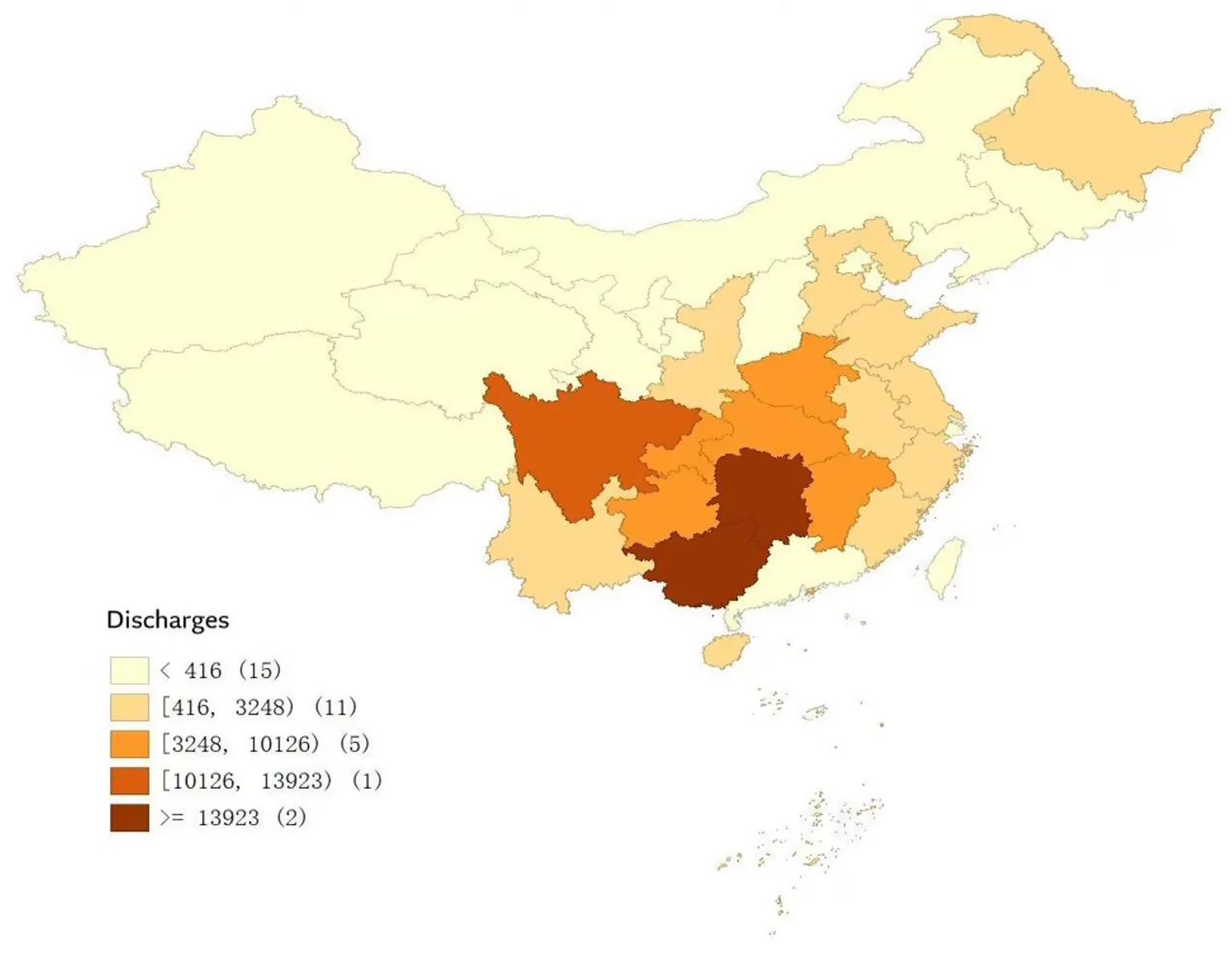
Distribution map of source provinces for cross-provincial medical care (2016–2025).

From 2016 to 2025, the top three source provinces for cross-provincial inpatients were Hunan Province, Guangxi Zhuang Autonomous Region, and Sichuan Province, accounting for 21.51, 20.52, and 14.93% of the total, respectively (Figure 3).

Global spatial autocorrelation analysis revealed a clustered pattern in the hospitalization volume of cross-provincial patients. The Global Moran’s I index was 0.248 (Z = 2.607, *p* < 0.05), confirming statistically significant positive spatial autocorrelation. This clustered pattern was consistent annually, with Moran’s I indices exceeding zero in each year from 2016 to 2025. Building on this, hotspot analysis identified Guizhou, Chongqing, and Hunan as statistically significant high-value clusters (hotspots) for cross-provincial hospitalizations (Table 2).

**Table 2:** Global Moran’s I indices and hotspot/coldspot areas for cross-provincial hospitalizations (2016–2025).

Year	Moran‘s I	Z value	*p*-value	Hotspot	Coldspot
2016	0.233	2.452	0.024	Yunnan, Guizhou, Chongqin, Hunan	
2017	0.248	2.626	0.014	Guizhou, Chongqin, Hunan	
2018	0.255	2.672	0.016	Guizhou, Chongqin, Hunan	
2019	0.263	2.748	0.014	Guizhou, Chongqin, Hunan	
2020	0.234	2.438	0.023	Guizhou, Chongqin, Hunan	
2021	0.243	2.513	0.019	Guizhou, Chongqin, Hunan	Xinjiang
2022	0.239	2.495	0.019	Guizhou, Chongqin, Hunan	Xinjiang
2023	0.250	2.909	0.013	Guizhou, Chongqin, Hunan	
2024	0.238	2.677	0.015	Guizhou, Hunan	
2025	0.252	2.816	0.012	Guizhou, Hunan	
2016–2025	0.248	2.607	0.018	Guizhou, Chongqin, Hunan	

### 3.5 Departmental distribution for cross-provincial inpatients

From 2016 to 2025, the Orthopedics and Traumatology Department was the primary admitting department for cross-provincial inpatients at this hospital, accounting for 49.25% of cases. This was followed by Internal Medicine (14.95%), Surgery (13.91%), and Neurology (4.74%) (Table 3). “Mobile Cabin Ward” appearing only in 2022: This is a true COVID-19 pandemic response, not a data recording issue. In 2022, during the peak of the COVID-19 pandemic, the hospital urgently activated a mobile cabin ward to treat mild cases and quarantine observation patients. After the pandemic subsided, the ward was decommissioned or placed on standby. Thus, its appearance only in 2022 reflects an authentic event of temporary medical resource allocation during a specific period. Its inclusion followed consistent case classification rules throughout the entire study period.

**Table 3:** Departmental distribution of hospitalizations for cross-provincial patients at the hospital (2016–2025).

Rank	Department	Discharges											PCT/%
		2016	2017	2018	2019	2020	2021	2022	2023	2024	2025	Total	
1	Orthopedics department	3,965	4,266	4,198	4,375	2,468	2053	1,407	2,906	3,766	4,008	33,412	49.25
2	Internal medicine department	1,170	1,154	1,159	1,377	787	549	478	1,006	1,174	1,286	10,140	14.95
3	Surgery department	1,267	1,132	1,053	1,039	701	518	415	890	1,203	1,217	9,435	13.91
4	Neurology department	330	327	391	422	247	193	134	352	425	396	3,217	4.74
5	Oncology department	147	180	171	222	119	90	119	376	506	423	2,353	3.47
6	Gynecology department	412	301	292	309	137	106	86	194	252	106	2,195	3.24
7	ENT department	238	234	254	240	121	105	55	130	148	88	1,613	2.38
8	Endocrinology department	131	144	149	193	101	89	57	178	155	187	1,384	2.04
9	Acupuncture department	158	136	149	144	54	54	48	145	163	175	1,226	1.81
10	Ophthalmology department	185	116	128	140	64	65	40	76	114	165	1,093	1.61
11	Stomatology department	124	149	157	116	76	58	40	114	159	74	1,067	1.57
12	Emergency department	25	38	44	50	6	9	0	10	31	18	231	0.34
13	GP department	0	0	0	0	0	0	0	57	55	81	193	0.28
14	ICU	16	20	17	22	7	1	4	9	14	8	118	0.17
15	Mobile cabin ward	0	0	0	0	0	0	117	0	0	0	117	0.17
16	Pain management department	0	0	0	0	0	0	0	0	9	29	38	0.06
17	Tanzhou cabin No. 2	0	0	0	0	0	0	3	0	0	0	3	0.00

### 3.6 Disease spectrum of cross-provincial inpatients

Table 4 details the disease spectrum of cross-provincial inpatients at this hospital from 2016 to 2025. Classified by the ICD-10 insurance version chapters, the three most prevalent disease systems were: (1) Injury, poisoning and certain other consequences of external causes (22,298 discharges), (2) Diseases of the musculoskeletal system and connective tissue (9,323 discharges), and (3) Factors influencing health status and contact with health services (8,359 discharges). The disease systems associated with higher average costs per hospitalization were Injury, poisoning and certain other consequences of external causes, Diseases of the musculoskeletal system and connective tissue, and diseases of the circulatory system.

**Table 4:** Analysis of the disease spectrum among cross-provincial inpatients at the hospital.

Rank	ICD-10 code	Systemic disease	Discharges	PCT/%	Avg. cost (CNY)
1	S00-T98	Injury, poisoning, and certain other consequences of external causes	22,298	32.87	30859.35
2	M00-M99	Diseases of the musculoskeletal system and connective tissue	9,323	13.74	22001.40
3	ZOO-Z99	Factors influencing health status and contact with health services	8,359	12.32	9222.32
4	K00-K93	Diseases of the digestive system	6,019	8.87	9747.14
5	I00-I99	Diseases of the circulatory system	4,650	6.85	17848.19
6	N00-N99	Diseases of the genitourinary system	3,602	5.31	11018.09
7	C00-D48	Neoplasms	3,044	4.49	17111.98
8	J00-J99	Diseases of the respiratory system	1913	2.82	11964.11
9	E00-E90	Endocrine, nutritional, and metabolic diseases	1,564	2.31	9071.10
10	G00-G99	Diseases of the nervous system	1,541	2.27	10501.37
11	A00-B99	Certain infectious and parasitic diseases	1,535	2.26	10946.27
12	O00-O99	Pregnancy, childbirth, and the puerperium	852	1.26	4133.40
13	H00-H59	Diseases of the eye and adnexa	815	1.20	8337.90
14	H60-H95	Diseases of the ear and mastoid process	670	0.99	8765.75
15	R00-R99	Symptoms, signs, and abnormal clinical and laboratory findings, not elsewhere classified	468	0.69	6795.69
16	Q00-Q99	Congenital malformations, deformations, and chromosomal abnormalities	346	0.51	17143.13
17	L00-L99	Diseases of the skin and subcutaneous tissue	317	0.47	8458.87
18	D50-D89	Diseases of the blood and blood-forming organs and certain disorders involving the immune mechanism	257	0.38	11761.82
19	F00-F99	Mental and behavioral disorders	140	0.21	7639.58
20	U00-U99	Codes for special purposes	122	0.18	2218.02

Regarding the top 20 diseases ranked by the number of discharges, the three most frequent conditions were removal of internal fixation device for fracture, lumbar disc herniation, and calcaneal fracture. Among these top 20 principal diagnoses, the three diseases associated with the highest average medical costs per hospitalization were fracture of the shaft of the tibia and fibula, lumbar spinal stenosis, and femoral neck fracture (Table 5).

**Table 5:** Top 20 diseases for cross-provincial hospitalizations at the hospital (by number of discharges).

Disease name	Discharges	PCT/%	Avg. cost (CNY)
Removal of internal fixation device for fracture	3,971	5.85	7390.61
Lumbar disc herniation	1,371	2.02	11810.60
Calcaneal fracture	1,079	1.59	33777.01
Mixed hemorrhoids	813	1.20	7648.78
Maintenance chemotherapy for malignant Tumor	737	1.09	13787.64
Lumbar disc herniation with radiculopathy	630	0.93	13356.81
Postoperative chemotherapy for malignant tumor	614	0.91	11571.26
Femoral neck fracture	583	0.86	38478.14
Chronic hepatitis B	565	0.83	7487.10
Chronic gastritis	547	0.81	6726.28
Patellar fracture	491	0.72	23124.44
Cerebral infarction	485	0.71	13078.99
Lumbar vertebral fracture	455	0.67	34021.68
Lacunar infarction	423	0.62	10116.68
Lumbar spinal stenosis	418	0.62	41291.65
Distal radius fracture	401	0.59	29176.49
Tibial and fibular shaft fracture	400	0.59	46339.16
Intertrochanteric fracture of femur	388	0.57	37112.90
Crush injury of finger	367	0.54	11879.52
Clavicular fracture	340	0.50	28017.51

### 3.7 Hospitalization costs for cross-provincial inpatients

**Figure 4:**
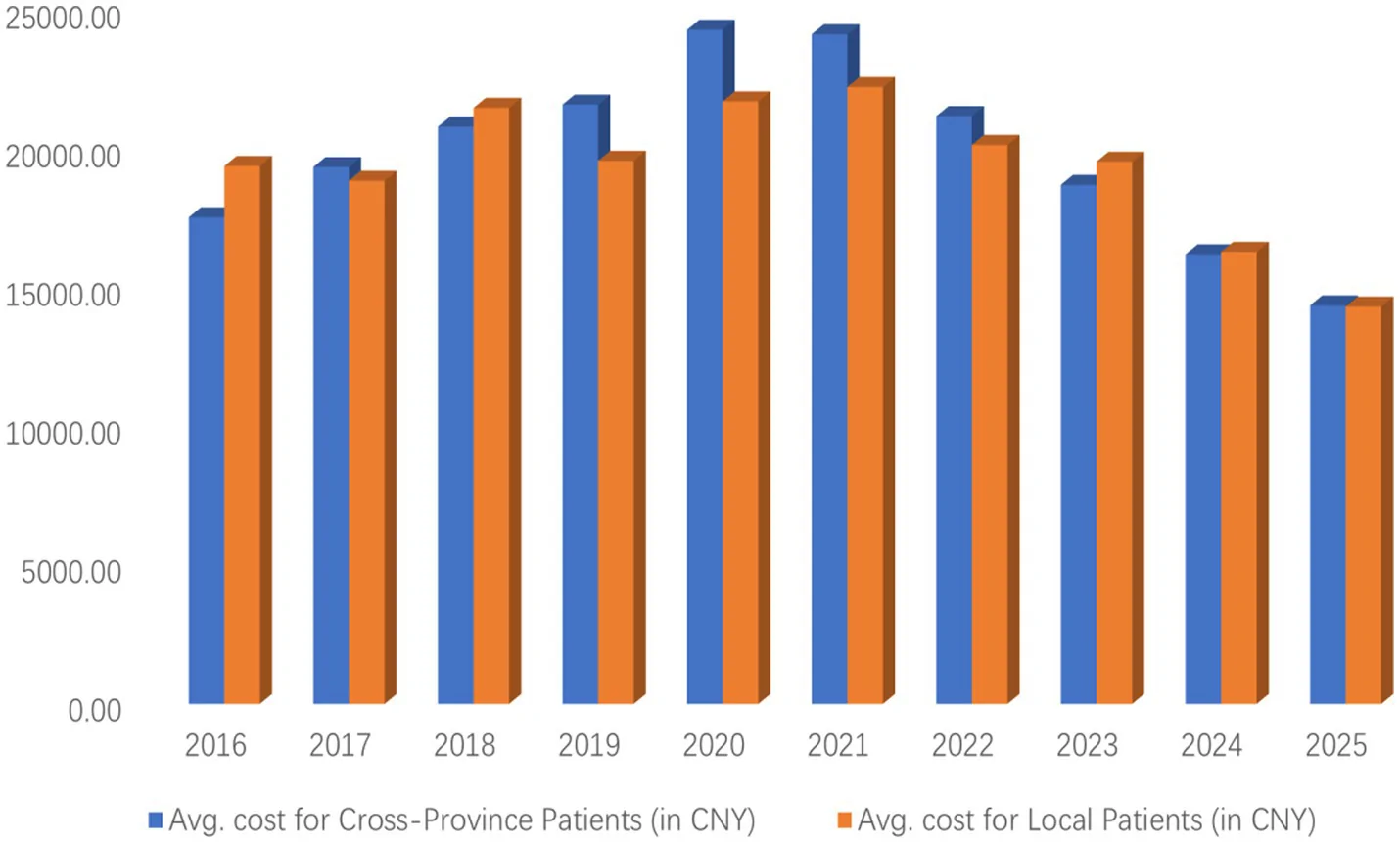
Average cost per hospitalization for cross-provincial versus local patients (2016–2025).

The comparison of average hospitalization costs per case between cross-provincial patients and local patients from 2016 to 2025 is presented in Figure 4. The Shapiro–Wilk test for normality was performed on the two groups of data, yielding a *p*-value > 0.05, indicating that the data met the assumption of normal distribution. The F-test was used to assess the homogeneity of variances, with *F* = 0.130 and *p* > 0.05, indicating that the homogeneity of variance assumption was satisfied. Based on the results of the normality test and the homogeneity of variance test, the conditions for applying the independent samples t-test were met. The t-test yielded t = 0.361 and *p* > 0.05, suggesting that the difference between the two groups was not statistically significant. In other words, there was no difference in average hospitalization cost per case between cross-provincial patients and local patients.

Based on the hospitalization medical record front sheet, costs are classified into ten major categories. An analysis of the average cost per hospitalization and its composition for cross-provincial patients from 2017 to 2019 revealed that medical supplies (consumables) constituted the largest share (36.03%), followed by treatment costs (17.46%) and diagnostic costs (16.04%). Despite its significant proportion, the share of medical supplies has demonstrated a year-on-year declining trend since 2021, following the hospital’s implementation of its first batch of centralized procurement for such supplies on February 1, 2021. Conversely, the proportions for both treatment and diagnostic costs have shown a consistent year-on-year increase during the same period (Table 6).

**Table 6:** Distribution of hospitalization cost categories for cross-provincial patients, 2016–2025.

Cost breakdown		2016	2017	2018	2019	2020	2021	2022	2023	2024	2025	Total
General medical service	Avg. cost (CNY)	1088.36	1428.95	1629.20	1569.31	1646.20	1619.17	1713.38	1516.71	1447.79	1473.07	1485.06
	PCT/%	6.21	7.39	7.83	7.27	6.78	6.71	8.09	8.11	8.94	10.26	7.71
Diagnostic service	Avg. cost (CNY)	2516.25	2760.51	2926.68	2968.16	3045.12	3198.51	3362.26	3657.46	3614.10	3181.20	3089.11
	PCT/%	14.35	14.27	14.07	13.74	12.54	13.25	15.87	19.55	22.31	22.15	16.04
Therapeutic service	Avg. cost (CNY)	2534.77	3014.10	3384.95	3375.03	3851.54	4032.51	3839.86	3520.64	3462.96	3492.46	3362.78
	PCT/%	14.46	15.58	16.27	15.63	15.85	16.71	18.13	18.82	21.38	24.32	17.46
Rehabilitation service	Avg. cost (CNY)	229.03	275.65	254.24	243.99	250.68	237.58	241.50	218.50	200.20	181.09	231.89
	PCT/%	1.31	1.42	1.22	1.13	1.03	0.98	1.14	1.17	1.24	1.26	1.20
Traditional Chinese medical service	Avg. cost (CNY)	1464.82	1639.49	1715.36	1686.86	1724.69	1962.83	2020.54	2099.59	2155.54	2000.05	1824.97
	PCT/%	8.35	8.47	8.25	7.81	7.10	8.13	9.54	11.22	13.31	13.93	9.48
Western medicine	Avg. cost (CNY)	1774.70	1999.88	1906.93	1787.35	1877.53	1717.88	1572.94	1289.91	1171.51	1097.44	1613.45
	PCT/%	10.12	10.34	9.17	8.28	7.73	7.12	7.43	6.90	7.23	7.64	8.38
Chinese medicine	Avg. cost (CNY)	591.92	643.98	676.79	628.74	545.18	665.05	694.45	624.38	600.06	635.98	627.91
	PCT/%	3.38	3.33	3.25	2.91	2.24	2.76	3.28	3.34	3.70	4.43	3.26
Blood and blood products	Avg. cost (CNY)	80.95	129.09	98.90	76.03	73.81	68.84	68.14	73.32	66.90	59.38	81.48
	PCT/%	0.46	0.67	0.48	0.35	0.30	0.29	0.32	0.39	0.41	0.41	0.42
Medical consumables	Avg. cost (CNY)	7251.82	7452.18	8202.58	9259.87	11277.48	10625.67	7668.53	5703.14	3481.53	2239.96	6936.70
	PCT/%	41.36	38.52	39.43	42.88	46.42	44.03	36.20	30.49	21.49	15.60	36.03
Others	Avg. cost (CNY)	0.62	1.92	4.72	1.42	0.45	2.90	0.07	1.52	0.22	0.00	1.43
	PCT/%	0.00	0.01	0.02	0.01	0.00	0.01	0.00	0.01	0.00	0.00	0.01

Grey relational analysis was performed using the average cost per hospitalization (X0) as the mother sequence and ten cost categories as child sequences: comprehensive medical services (X1), diagnostics (X2), treatment (X3), rehabilitation (X4), TCM services (X5), western medicine (X6), Chinese herbal medicine (X7), blood and blood products (X8), medical supplies/consumables (X9), and others (X10). The resulting relational coefficients are presented in Table 7. The ranking of these cost categories by their degree of influence on the total hospitalization cost, from highest to lowest, is as follows: medical supplies/consumables > treatment > diagnostics > TCM services > western medicine > comprehensive medical services > Chinese herbal medicine > rehabilitation > blood and blood products > others.

**Table 7:** Results of grey relational analysis on hospitalization costs for cross-provincial patients (2016–2025).

Item	Correlation r	Rank
Medical consumables (X9)	0.912	1
Therapeutic service (X3)	0.796	2
Diagnostic service (X2)	0.788	3
Traditional Chinese medical service (X5)	0.754	4
Western medicine (X6)	0.747	5
General medical service (X1)	0.744	6
Chinese medicine (X7)	0.723	7
Rehabilitation service (X4)	0.714	8
Blood and blood products (X8)	0.711	9
Others (X10)	0.709	10

## 4 Discussion and recommendations

This study, based on ten-year longitudinal inpatient data from Foshan Hospital of Traditional Chinese Medicine spanning 2016 to 2025, systematically analyzed the spatiotemporal distribution, disease spectrum, cost structure, and influencing factors of cross-provincial inpatients. The following discussion is organized around three levels: implications for patients and access to medical services, implications for healthcare institution management, and implications for regional and national health policy.

### 4.1 Implications for patients and access to medical services

This study found that the source regions of cross-provincial inpatients exhibited significant spatial clustering, with hotspots concentrated in Guizhou, Chongqing, and Hunan provinces, predominantly in conditions related to orthopedics and traumatology. This spatial pattern reflects the underlying logic of cross-provincial healthcare utilization: patients actively seeking high-quality specialist medical resources, while simultaneously exposing structural deficiencies in the corresponding specialist services in their home provinces.

Patient mobility serves both as an indicator of high-performance medical centers and as a direct reflection of interregional healthcare resource imbalances. In 2024, the number of cross-regional medical visits nationwide reached 397 million, with total expenditures of 786.774 billion RMB, both showing an increasing trend. The spatial pattern of cross-provincial healthcare utilization indicates that medical resources are highly concentrated in economically developed and provincial capital cities, with geographical disparities significantly driving patient flows toward high-resource areas. Studies have shown that for every 1% increase in the geographic disparity of high-quality medical resources, cross-city medical expenditures increase by approximately 0.10%. From the perspective of patient outflow provinces, Guangxi, Hunan, and Sichuan are typical “patient-exporting” regions, where the demand for out-of-region medical care is driven both by preferences for higher-quality services and by the relative inadequacy of local high-quality medical resources.

In this study, cross-provincial patients were predominantly middle-aged males aged 31–60 years (accounting for 62.94%), with injuries and poisoning as the primary disease categories, consistent with related studies (25–31). As the backbone of social production, this population has greater exposure to outings, higher work intensity, and elevated occupational risks, making their healthcare choices driven by labor protection needs (32, 33). Notably, Guizhou, as one of the hotspot provinces, is closely associated with the hospital’s cross-regional medical assistance and cooperation missions, suggesting that pairing assistance programs have a bidirectional effect in building brand reputation and attracting patients—while enhancing the medical service capacity of partner provinces, they also channel critically ill patients to the assisting hospital to a certain extent.

Reflections from the perspective of health equity. Although cross-provincial healthcare utilization reflects the protection of patients’ right to choice, it also implies that patients with better economic conditions or stronger information access are able to “vote with their feet” for superior services, while vulnerable groups may remain in their local areas due to information barriers and financial constraints. Existing research indicates that income disparities and differences in medical resources significantly influence patient mobility, with high-income regions often providing higher-quality services, thereby attracting patients from lower-income areas. In this study, the proportion of self-payment among cross-provincial patients remained relatively high (55.60%), suggesting that financial burden may constitute an important constraint on healthcare choices for some patients (34, 35). Constrained by the use of retrospective administrative data, this study was unable to incorporate socioeconomic variables such as patient income and education level, limiting an in-depth understanding of health equity issues in cross-provincial healthcare utilization. Future research should employ mixed methods including questionnaires and interviews to obtain first-hand information on patient decision-making, so as to more comprehensively reveal the social determinants of cross-provincial healthcare utilization.

### 4.2 Implications for healthcare institution management

The findings of this study have direct management implications for healthcare institutions that receive cross-provincial patients.

Discharge time patterns and resource allocation. Circular distribution analysis revealed that the discharge peak for cross-provincial patients was concentrated around August 17 (during the summer vacation period), suggesting that patients tend to utilize holiday time windows for medical treatment. This information can be used to optimize bed turnover planning and human resource allocation at settlement windows. Meanwhile, the length of hospital stay was predominantly 0–10 days (accounting for 70.72%), which is closely associated with the hospital’s implementation of the Enhanced Recovery After Surgery (ERAS) concept in orthopedics and the day surgery model. Removal of fracture internal fixation devices ranked first among cross-provincial disease categories; this type of surgery inherently features a short hospital stay, and with the implementation of the day surgery model, the hospital stay has been compressed from the traditional 4 days to within 24 h. For cross-provincial patients, the dramatic reduction in hospital stay directly lowers the indirect costs of out-of-town care (accommodation, accompanying family member expenses, and work absenteeism), making a “dedicated trip for hardware removal” an economically rational choice (36–39).

Cost structure control. Grey relational analysis revealed that material costs had the highest degree of association with per capita hospitalization costs and remained the primary factor influencing patient hospitalization expenses. Following the State Council’s proposal of the centralized procurement policy for medical consumables in 2019 (40), which was fully institutionalized in 2021 (41), the proportion of material costs at this hospital has declined year by year since 2021, playing a positive role in reducing patient burdens. However, the relational analysis suggests that material cost control remains the core focus of cost containment, and the hospital should continue to advance the implementation of centralized procurement policies and optimize material usage management.

Hospital “siphon effect” and policy synergy. This study found that although self-payment remained the primary payment method during 2016–2025 (accounting for 55.60%), the proportion of self-payment showed a significant downward trend, while the proportion of direct settlement increased correspondingly. Even during the early period when settlement was inconvenient and the self-payment rate was high, the hospital still attracted a large number of cross-provincial patients, fully demonstrating the brand resilience of the National Clinical Key Specialty in Orthopedics and Traumatology. With the hospital’s implementation of cross-provincial direct settlement for medical expenses in August 2017 and the continuous optimization of national cross-provincial healthcare policies, this siphon effect is expected to further intensify. However, since removal of fracture internal fixation devices ranks first among cross-provincial disease categories and this condition is not a critical or complicated illness, it reflects a failure to effectively achieve the hierarchical diagnosis and treatment goal of “serious illnesses do not leave the province.” In February 2026, the National Healthcare Security Administration initiated the establishment of a medical imaging cloud network for cross-provincial instant image retrieval, aiming to achieve barrier-free sharing and second-level retrieval of cross-provincial imaging data. The hospital can leverage this platform to enable rapid retrieval of patients’ off-site imaging data, avoid duplicate examinations, and reduce unnecessary cross-provincial physical travel through telemedicine and integrated medical alliance platforms.

### 4.3 Implications for regional and national health policy

The findings of this study have reference value for health policy formulation at a broader level.

Dynamic balance between cross-provincial healthcare utilization and hierarchical diagnosis and treatment. On the surface, cross-provincial healthcare utilization appears to contradict the “primary care first” principle of hierarchical diagnosis and treatment. However, a deeper analysis reveals that most cross-provincial healthcare utilization represents rigid demand (acute, critical, or severe conditions, or healthcare for the floating population), which does not conflict with the “upward referral of acute and severe cases” principle of hierarchical diagnosis and treatment. In 2024, cross-provincial inpatient direct settlement cases accounted for only approximately 4.9% of the insured population, exerting a limited systemic impact on the hierarchical diagnosis and treatment system. The primary challenge currently facing hierarchical diagnosis and treatment is not restricting patients’ right to choose cross-regional healthcare, but rather how to build a continuous health management system adapted to the characteristics of the floating population. The case of Chongqing exiting the hotspot list in 2024–2025 is particularly illustrative—the construction of the Jiangsu Province People’s Hospital Chongqing Hospital (a National Regional Medical Center) and the policy breakthrough of direct settlement for cross-provincial medical visits without prior registration in the Chengdu-Chongqing region have “intercepted” patients who would otherwise have flowed to Foshan within the Chengdu-Chongqing medical circle, validating the effectiveness of high-quality medical resource expansion and medical insurance policy synergy.

Policy effects of National Regional Medical Centers. Since the issuance of the “Pilot Work Plan for the Construction of Regional Medical Centers” by the National Development and Reform Commission and other departments in 2019, 125 National Regional Medical Center construction projects have been implemented nationwide, with over 1,400 diagnostic and treatment technologies applied in recipient provinces, significantly reducing cross-provincial and cross-regional healthcare utilization. National Regional Medical Centers have now covered all provinces except Beijing, Shanghai, and Tianjin, with projects in the central and western regions accounting for over 75%, primarily targeting diseases with high out-of-region referral rates such as oncology, neurology, cardiovascular diseases, and trauma. The phenomenon of Chongqing exiting the hotspot list observed in this study is a vivid manifestation of this policy effect—the improvement and expansion of local medical resources in Chongqing directly reduced the patient base that needed to travel to Foshan for medical care. Since the Jiangsu Province People’s Hospital Chongqing Hospital began construction in 2022, it has introduced 284 new technologies over three years and treated over 460,000 patients from outside the district. The Chengdu-Chongqing region also took the lead nationally in achieving direct settlement for cross-provincial medical visits without prior registration in 2022.

Policy directions for digital transformation. Digital technology holds significant potential in reducing geographical barriers. In February 2026, the National Healthcare Security Administration took the lead in establishing a medical imaging cloud network for cross-provincial instant image retrieval, requiring tertiary-level designated medical institutions with substantial cross-provincial healthcare service volumes to participate in the application. Upon selection, participating institutions can achieve second-level cross-provincial retrieval of patients’ nationwide medical imaging data. As a tertiary hospital that has ranked first among prefecture-level TCM hospitals nationwide for 12 consecutive years and handles a large volume of cross-provincial healthcare services, this hospital meets the application criteria. Joining this network would extend its imaging retrieval coverage to 24 provinces that have completed medical imaging cloud deployment, effectively reducing duplicate examinations and alleviating patients’ financial burden. Meanwhile, teleconsultation and AI-assisted diagnostic tools can extend high-quality specialist services to hotspot source provinces, reducing unnecessary cross-provincial physical travel at the source (42, 43).

Construction of regional medical networks. The spatial pattern of cross-provincial healthcare utilization suggests that establishing regional specialist alliances and cross-provincial referral green channels with hotspot source provinces (Guizhou, Chongqing, and Hunan) is of practical urgency. In 2024, Chenzhou City established medical insurance work liaison points at four Foshan hospitals, including Foshan Hospital of Traditional Chinese Medicine, to provide direct settlement service guarantees for cross-provincial migrant workers. This local exploration offers a replicable model for cross-provincial medical insurance coordination. It is recommended that such practices be further promoted, with medical insurance service windows established in major outflow areas of hotspot source provinces, facilitating the transformation from a model of “patients traveling across provinces” to one of “information flowing across provinces and resources allocated across regions”.

### 4.4 Transferability of the findings

The applicability of the findings of this study to other contexts depends on the similarity of specific circumstances. As this hospital has ranked first among prefecture-level TCM hospitals nationwide for 12 consecutive years, when transferring the findings to other tertiary hospitals, it is necessary to consider whether the target hospital possesses similar national-level specialty advantages. If it is a general hospital or a hospital specializing in other disciplines, the disease composition and spatial patterns of cross-provincial healthcare utilization may differ significantly. When transferring the findings to other regions of China, local geographical conditions must be considered—transportation convenience to neighboring provinces and interprovincial medical resource disparities both influence the specific patterns of cross-provincial healthcare utilization. From an international comparative perspective, healthcare systems with high patient mobility (such as among European Union member states) face similar policy challenges: how to promote balanced development of service capacity across regions while safeguarding patients’ freedom of choice. Spatial analysis of patient mobility can provide important references for health policy formulation, and the layout of National Regional Medical Centers should prioritize covering areas with concentrated patient outflows.

## 5 Limitations

First, the representativeness constraint of the single-center sample. The hospital has national-level specialty advantages in orthopedics and traumatology, and its cross-provincial patients are predominantly composed of elective orthopedic surgeries, which systematically differs from general hospitals or other specialty hospitals. Therefore, generalization of the study’s conclusions to other types of healthcare institutions should be approached with caution.

Second, the inherent limitations of retrospective administrative data. Cross-provincial patients were defined as inpatients whose registered domicile and current address were both outside Guangdong Province, which excluded the floating population whose registered domicile is outside Guangdong but who reside in Guangdong on a long-term basis. This may underestimate the population size actually affected by interprovincial medical resource allocation and also fails to capture the healthcare-seeking characteristics of “migratory” patients such as older adults relocating to live with their children.

Third, the absence of key sociodemographic variables. Due to data availability constraints, variables such as income, education level, urban/rural origin, and the actual distance from the patient’s residence to the hospital could not be included, limiting an in-depth analysis of health equity issues in cross-provincial healthcare-seeking behavior.

Fourth, the lack of patients’ subjective perspectives. This study is entirely based on a quantitative analysis from the healthcare institution’s perspective and does not incorporate patients’ perceptions and experiences. Cross-provincial healthcare-seeking behavior involves multiple factors, including patients’ perceptions of technical competence, risk–benefit trade-offs, and family support. Future research may employ questionnaires, interviews, or mixed methods to reveal the real reasons behind patients’ choice of out-of-province medical care from the patient perspective.

Fifth, the cost analysis only covers direct medical expenses and does not include indirect costs such as transportation, accommodation, accompanying family member expenses, and economic losses due to work absenteeism, resulting in an incomplete characterization of patients’ true economic burden.

In summary, the aforementioned limitations do not negate the value of this study but rather suggest that the research conclusions should be understood within specific boundary conditions. A more comprehensive elaboration of limitations helps enhance the credibility of the study and points the way for future multi-center, multi-method research designs.

## 6 Conclusion

This study, based on ten-year inpatient data from Foshan Hospital of Traditional Chinese Medicine (2016–2025), systematically analyzed the mobility patterns and influencing factors of cross-provincial patients from multiple dimensions, including spatial distribution, temporal rhythms, cost structure, and disease spectrum.

### 6.1 Major scientific contributions

First, a multidimensional integrated analytical framework was constructed. Spatial clustering analysis, discharge time periodicity identification, and cost structure correlation ranking were incorporated into a unified system, revealing the systematic characteristics of cross-provincial healthcare utilization in terms of source regions, temporal patterns, and cost composition, thereby providing a replicable methodological reference for functional assessment of regional medical centers.

Second, the “dual-layer demand structure” of regional medical centers was identified. Cross-provincial and local patients exhibited markedly different response patterns to external shocks: local demand served as the “ballast” of services, while cross-provincial patient volume functioned as a “sensitive barometer” of external attractiveness. During the pandemic, cross-provincial patients showed greater declines and slower recovery than local patients, offering a novel theoretical explanation for understanding the vulnerability of cross-regional allocation of high-quality medical resources from the perspective of demand elasticity.

Third, regional spatial patterns with planning value were identified. Hotspots were concentrated in Guizhou, Chongqing, and Hunan provinces, predominantly featuring diseases related to national key specialties such as orthopedics and traumatology. This delineated the actual radiation boundary of the regional TCM orthopedic medical center, providing evidence-based geographical decision-making support for optimizing key specialty distribution and regional medical center construction.

### 6.2 Implications for public health and hospital management

(1) Reducing regional medical disparities: Service capacity should be extended to hotspot source provinces through specialty alliances and paired assistance programs, reducing unnecessary cross-provincial patient flows at the source. (2) Enhancing access to specialized services: High-quality resources should be disseminated through teleconsultation and expert outreach programs. (3) Optimizing reimbursement mechanisms: Cost correlation analysis identified key cost drivers, providing a basis for adjusting cross-provincial direct settlement standards. (4) Promoting interregional medical collaboration: Cross-provincial referral green channels and mutual recognition of examination results should be established, facilitating the transition from “patients traveling across provinces” to “resources flowing across regions”.

### 6.3 Future research directions

Future research should incorporate multi-center comparisons, integrate socioeconomic variables, introduce patients’ subjective perspectives, and evaluate the actual effectiveness of telemedicine in reducing cross-provincial physical travel for medical care.

In summary, this study provides a scientific basis for optimizing disciplinary layout, improving cross-provincial healthcare management, and advancing refined hospital management.

## Data Availability

The data analyzed in this study is subject to the following licenses/restrictions: the data analyzed in this study are subject to restrictions due to the nature of the data containing potentially sensitive patient information. The dataset contains personal health information of hospitalized patients, and access is strictly controlled to protect patient privacy in accordance with the ethical standards and the confidential commitments made during the informed consent process. Therefore, the raw data cannot be made publicly available. Requests to access these datasets should be directed to Luqing Zeng, 13535540038@163.com.
