# Supplementary material for: Analysis of cross-provincial healthcare utilization among hospitalized patients in a tertiary Chinese medicine hospital
Source: Front Public Health. 2026 Aug 17;14:1818250. doi: 10.3389/fpubh.2026.1818250 (PMC13528336; doi:10.3389/fpubh.2026.1818250)
Supplement: Supplementary file 1 [file Data_Sheet_1.pdf]

Supplementary Information

Analysis of Cross-provincial Healthcare Utilization Among Hospitalized Patients in a Tertiary Chinese Medicine Hospital

Luqing Zeng[1,*], Junying Wang[2], Hua Zhang[4], Jian Liang[2],Hanhua Bai[3],Yanbin Yang[1,*]

[1] Medical Records and Statistics Department, Foshan Hospital of Traditional Chinese Medicine, Foshan, Guangdong, China

[2] Social Health Insurance Department, Foshan Hospital of Traditional Chinese Medicine, Foshan, Guangdong, China

[3] Department of Science and Education, Foshan Hospital of Traditional Chinese Medicine,Foshan, Guangdong, China

[4] Department of Medical Quality Management, Foshan First People's Hospital, Foshan, Guangdong, China

* Correspondence to: 13535540038@163.com and 18929946709@189.com

Note1. Comparison of the Decline in Hospitalizations Between Local Patients and Cross-Provincial Patients During the Pandemic

Cross-provincial patients showed a steeper decline (20%) over a longer period (three years), while local patients declined only in the first year of the pandemic. This suggests that cross-provincial volume is a sensitive measure of a regional medical center's "attractiveness elasticity" — these patients are the first to be displaced by external disruptions (policy, transportation, public health). Local patients, by contrast, act as the hospital's "ballast," with demand largely resilient to external shocks, underscoring the essential nature of local care. Cross-provincial care exhibits high demand elasticity, whereas local care exhibits low demand elasticity with strong rigidity

and resilience (Figs.S1 and S2).

Fig.S1 Trends in cross-provincial inpatient admissions, 2016–2025

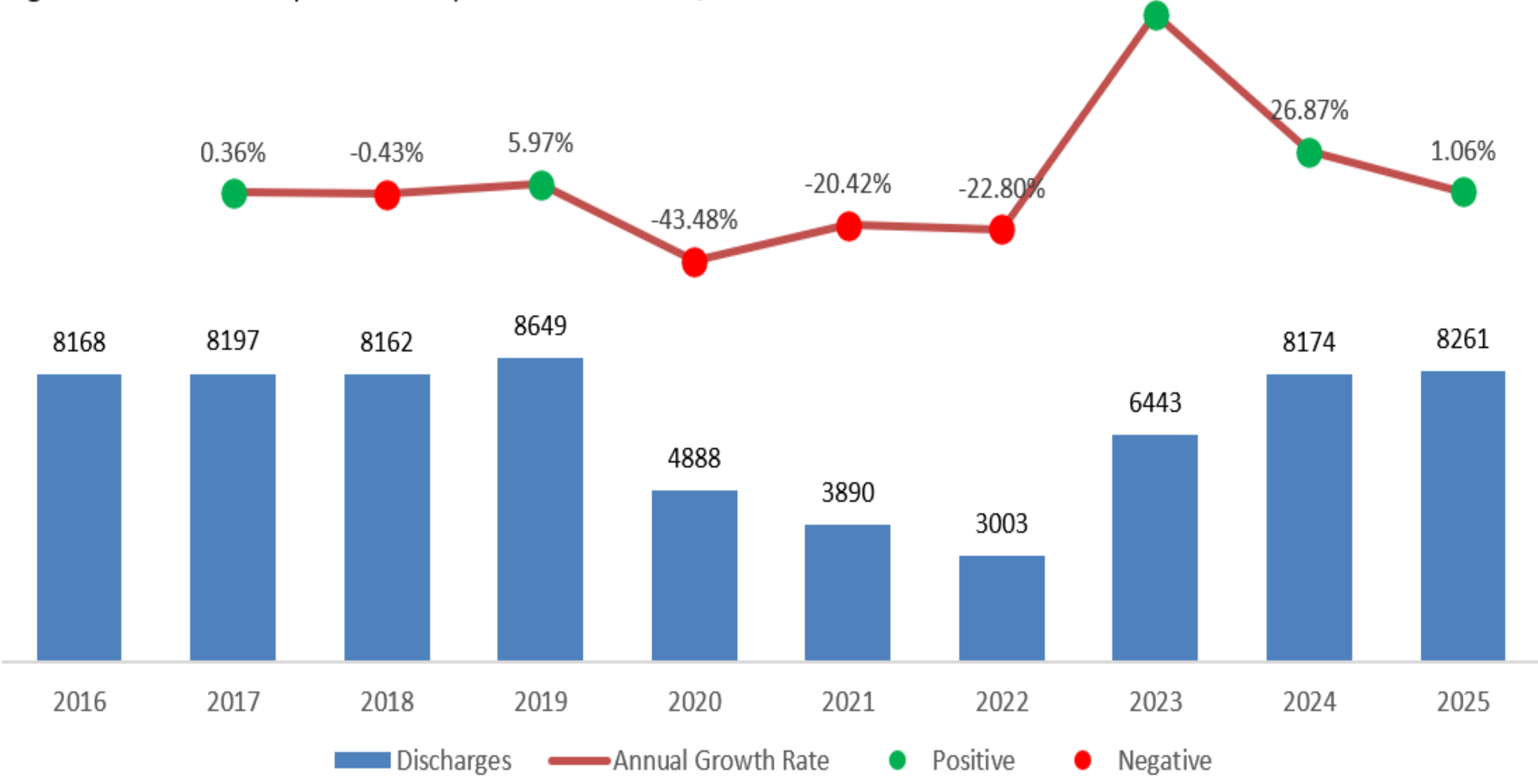

Fig.S2 Trends in local inpatient admissions, 2016–2025

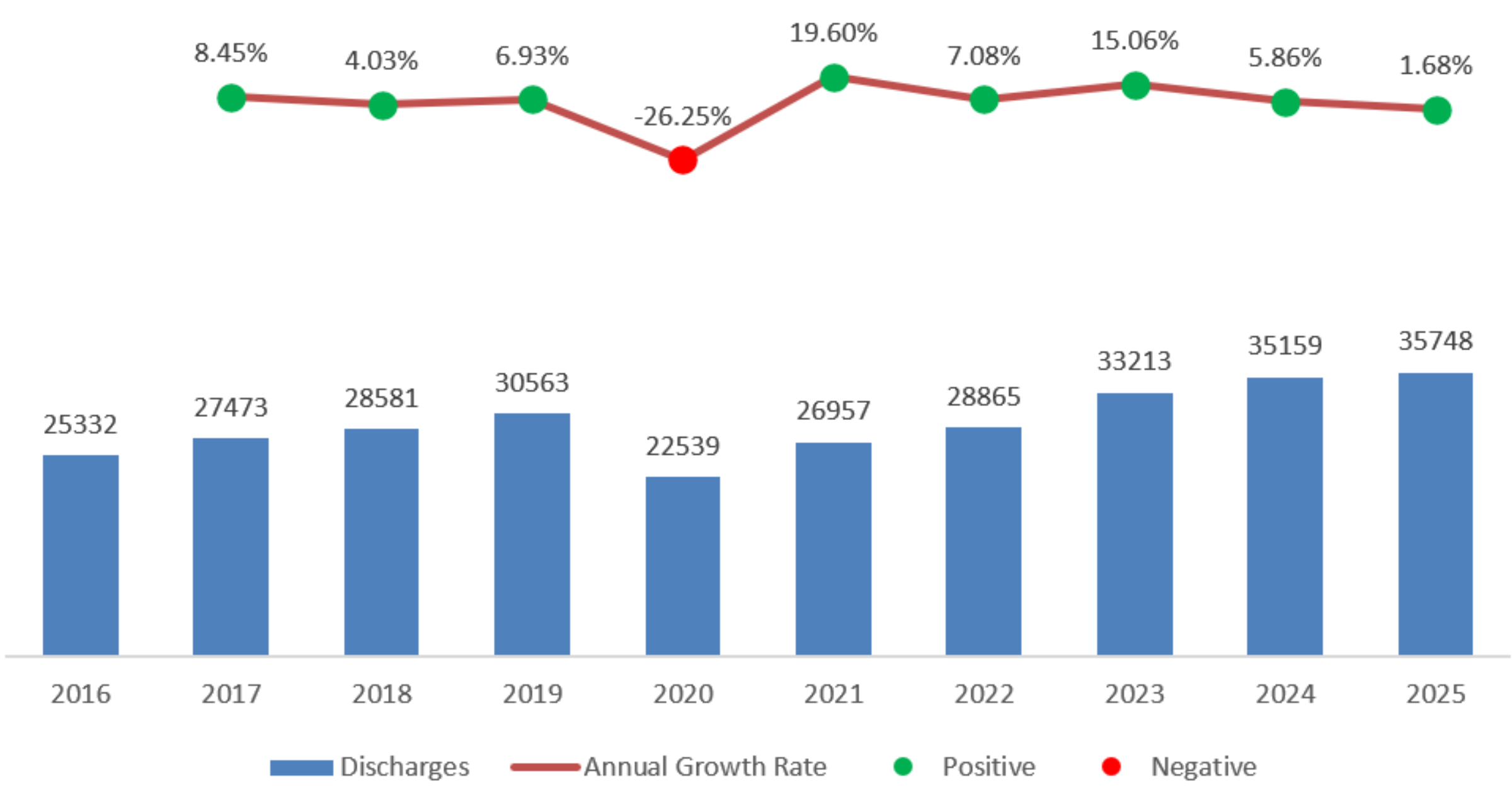

Note2. Total cost and annual growth rate by year, 2016–2025

The annual growth rate of hospitalization costs for cross-provincial inpatients remained relatively stable between 2017 and 2019. During the COVID-19 pandemic, costs fell by approximately 20–30%. A sharp rebound occurred in 2023, with a

year-on-year increase of 89.48%, followed by a decline of 10.42% in 2025. (Figs.S3).

Fig.S3 Trends in hospitalization costs of cross-provincial patients, 2016–2025

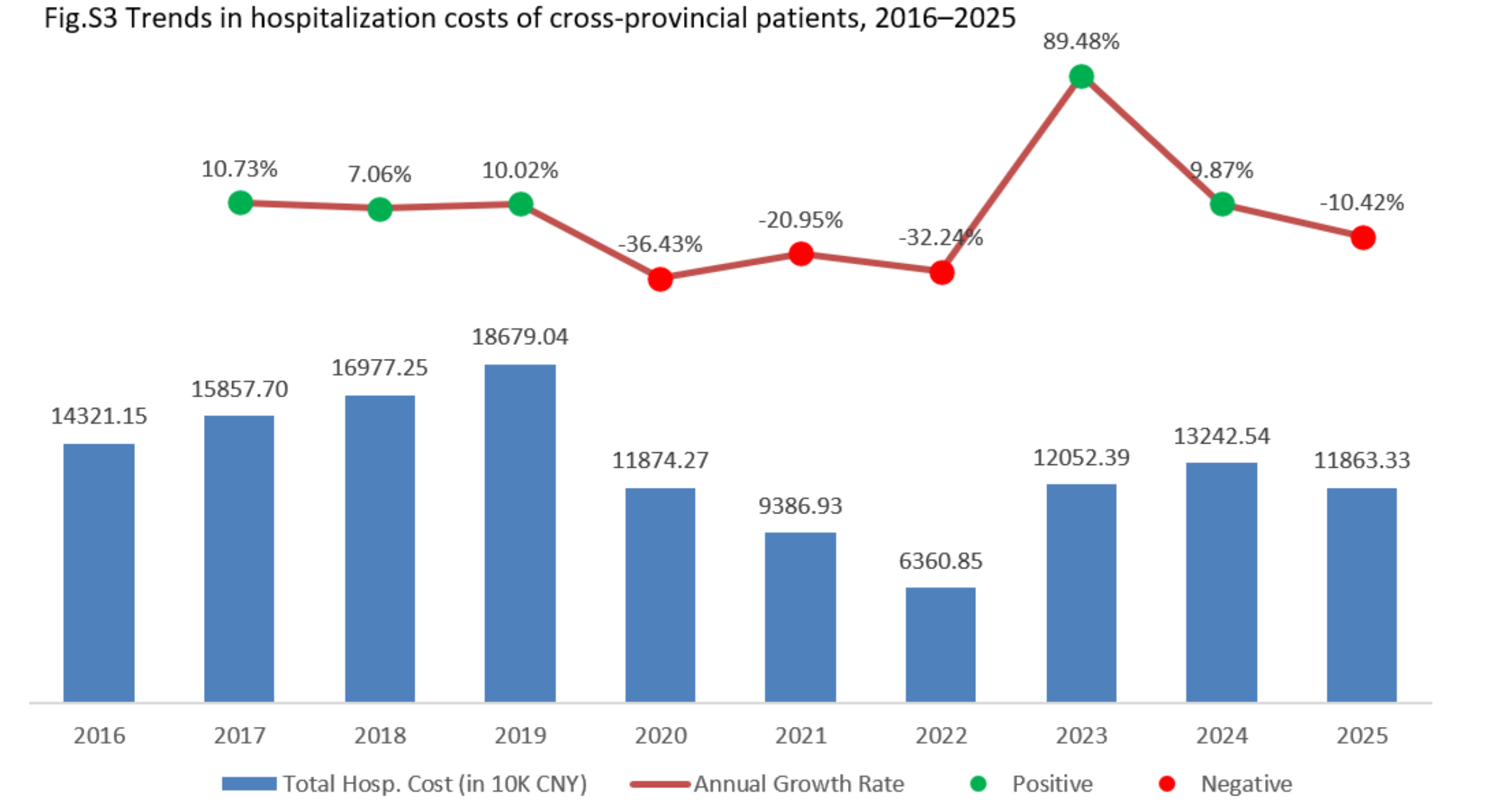

Note3. Post-pandemic provincial recovery patterns in cross-provincial care

The provinces that recovered better after the pandemic were Hunan, Guangxi, Sichuan, Jiangxi, and Hubei. (Figs.S4).

Fig.S4 Distribution of Cross-Provincial Patient Sources by Region (Percentage), 2022–2025

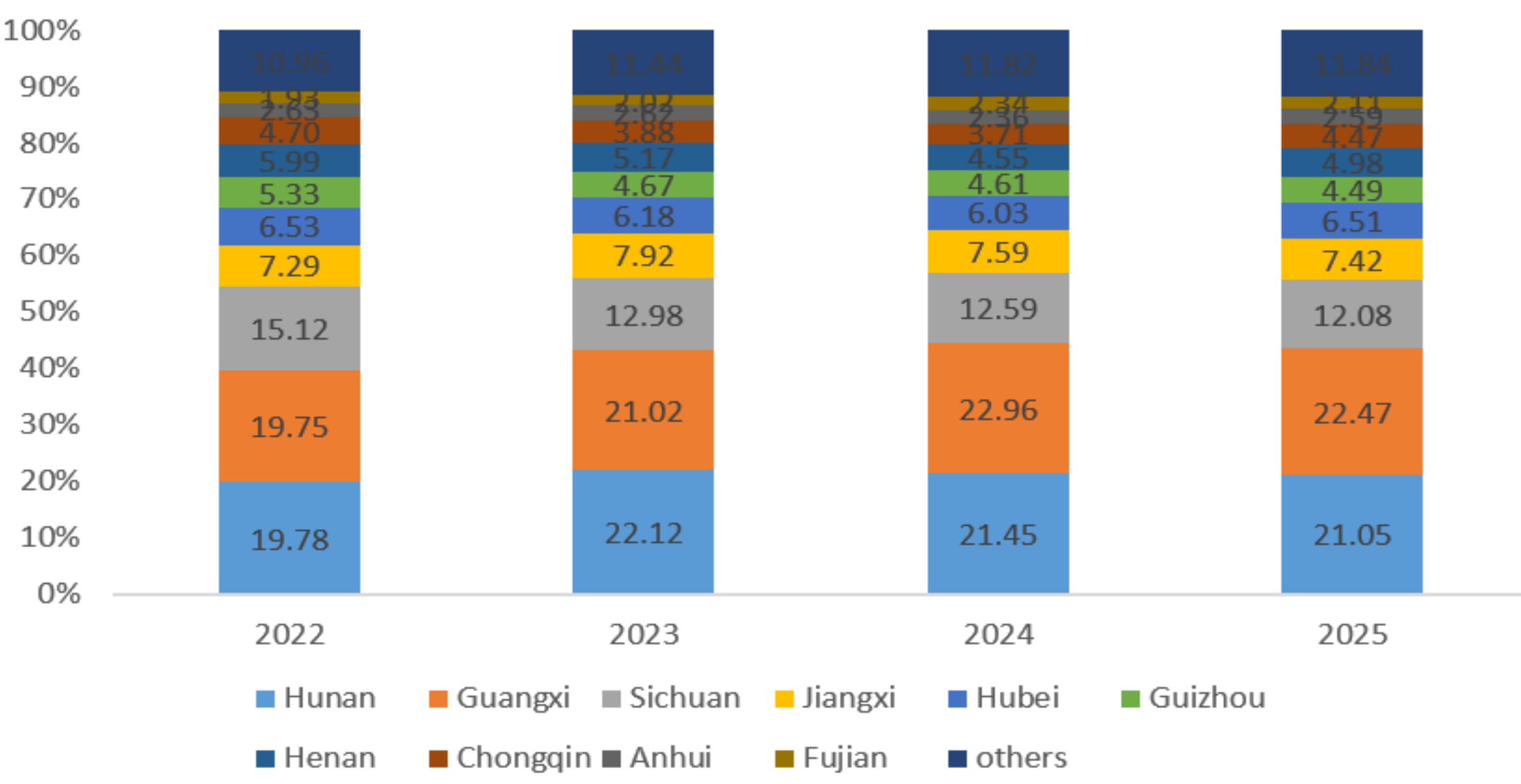

Note4. Proportions of Demographic and Basic Characteristics Among Cross-Provincial Hospitalized Patients, 2016–2025

With the advancement of the cross-provincial settlement policy, the proportion of patients aged 46–60 years has increased year by year, while the proportion of self-paying patients has decreased annually, falling from 87.07% in 2016 to 10.05% in 2025. (Table 1).

Table 1. Trends in demographic composition of cross-provincial inpatients(2016–2025)

Item	PCT/%										
	2016	2017	2018	2019	2020	2021	2022	2023	2024	2025	Total
Sex											
Male	64.52	64.88	64.58	62.90	64.18	65.60	64.34	60.28	59.41	60.96	62.94
Female	35.48	35.12	35.42	37.10	35.82	34.40	35.66	39.72	40.59	39.04	37.06
Age											
0-18 years	6.35	5.44	4.83	5.49	4.38	4.14	3.50	4.86	5.14	4.45	5.03
19-30 years	19.68	16.36	13.94	13.25	10.90	10.82	8.69	9.78	9.54	8.59	12.48
31-45 years	23.73	35.74	35.21	31.34	32.02	30.05	29.50	26.73	26.22	26.03	31.33
46-60 years	20.27	32.05	34.60	36.59	39.57	40.64	43.02	40.38	41.35	41.65	37.01
60+ years	8.75	10.41	11.42	13.32	13.13	14.34	15.28	18.25	17.75	19.27	14.14
Marital Status											
Married	84.90	86.41	80.57	76.07	76.90	78.53	79.99	82.26	83.55	84.46	81.81
Single	14.50	12.74	12.85	13.45	12.15	12.90	10.26	13.97	14.68	13.82	13.39
Other	0.02	0.09	5.22	9.20	8.84	6.71	7.43	2.67	0.99	0.87	3.64
Divorced	0.27	0.45	0.82	0.59	1.00	0.87	1.20	0.56	0.39	0.48	0.60
Widowed	0.31	0.32	0.54	0.69	1.10	0.98	1.13	0.54	0.39	0.36	0.56
Insurance_Type											
Social Basic Medical Insurance	12.90	13.09	17.54	24.21	30.97	34.99	45.79	68.35	53.51	46.24	33.17
Commercial Health Insurance	0.00	0.00	0.00	0.00	0.12	1.52	1.57	0.02	0.02	0.00	0.17
Out-of-pocket	87.07	86.91	82.46	75.75	68.06	62.78	49.15	16.30	13.09	10.05	55.60
Other	0.02	0.00	0.00	0.03	0.84	0.72	3.50	15.33	33.37	43.71	11.07
Length of Hospital Stay											
0-10 days	65.95	64.99	66.21	69.13	70.64	71.26	70.83	75.79	76.52	77.32	70.72
11-20 days	24.13	24.01	24.38	23.18	21.87	21.77	21.81	18.89	18.88	18.42	21.80
21-30 days	5.51	6.37	6.14	5.19	4.75	4.37	4.90	3.52	3.01	2.94	4.70
31-40 days	2.25	2.17	1.57	1.19	1.47	1.26	1.07	0.99	0.89	0.74	1.39
40+ days	2.15	2.46	1.70	1.31	1.27	1.34	1.40	0.81	0.70	0.58	1.39

Note5. Analysis of Length of Hospital Stay for the Top 20 Diseases Among Cross-Provincial Inpatients (Ranked by Number of Discharges)

Among the top 20 cross-provincial diseases, removal of fracture internal fixation devices and chronic gastritis had the shortest average length of hospital stay, while

lumbar vertebral fracture and tibial and fibular shaft fracture had the longest average length of hospital stay. (Table 2).

Table 2. Length of hospital stay for the top 20 diseases among cross-provincial inpatients at the hospital (by number of discharges)

Disease Name	Avg. LOS（DAY)
Removal of Internal Fixation Device for Fracture	5.40
Lumbar Disc Herniation	10.65
Calcaneal Fracture	12.10
Mixed Hemorrhoids	6.98
Maintenance Chemotherapy for Malignant Tumor	8.75
Lumbar Disc Herniation with Radiculopathy	10.34
Postoperative Chemotherapy for Malignant Tumor	6.54
Femoral Neck Fracture	10.90
Chronic Hepatitis B	6.68
Chronic Gastritis	5.38
Patellar Fracture	10.96
Cerebral Infarction	9.18
Lumbar Vertebral Fracture	15.54
Lacunar Infarction	7.24
Lumbar Spinal Stenosis	12.16
Distal Radius Fracture	9.67
Tibial and Fibular Shaft Fracture	15.20
Intertrochanteric Fracture of Femur	12.61
Crush Injury of Finger	8.40
Clavicular Fracture	8.84

Note6. Analysis of Per Capita Hospitalization Cost Differences Among Cross-Provincial Patients by Age Group

The analysis shows that the per capita hospitalization cost increases with patient age. (Table 3).

Table 3. Age-Related Variations in Per Capita Inpatient Expenditure Among Cross-Provincial Patients

Age	Avg.cost(CNY)
0-18 years	14565.85
19-30 years	17604.25
31-45 years	18830.35
46-60 years	20093.31
60+ years	21126.53

Note7. Comparative Analysis of Per Capita Inpatient Expenditure Between Insured Patients and Self-Paying Patients

The per capita inpatient expenditure of self-paying patients exceeded that of insured patients (Table 4).

Table 4. Analysis of Per Capita Hospitalization Cost Differences Between Insured and Self-Paying Patients

Insurance_Type	Discharges	Avg.cost(CNY)
insured patients	30122	15929.88
self-paying patients	37713	21910.60

Note8. Analysis of Per Capita Hospitalization Cost Differences Across Major cross-provincial Patient Source Regions

Among the top 10 cross-provincial patient source provinces, Guizhou (RMB 20,670.34) and Guangxi (RMB 20,664.24) had the highest per capita hospitalization costs (see details in Figs.S5).

Fig S5 Per Capita Cost Variation Among Major cross-provincial Patient Origins

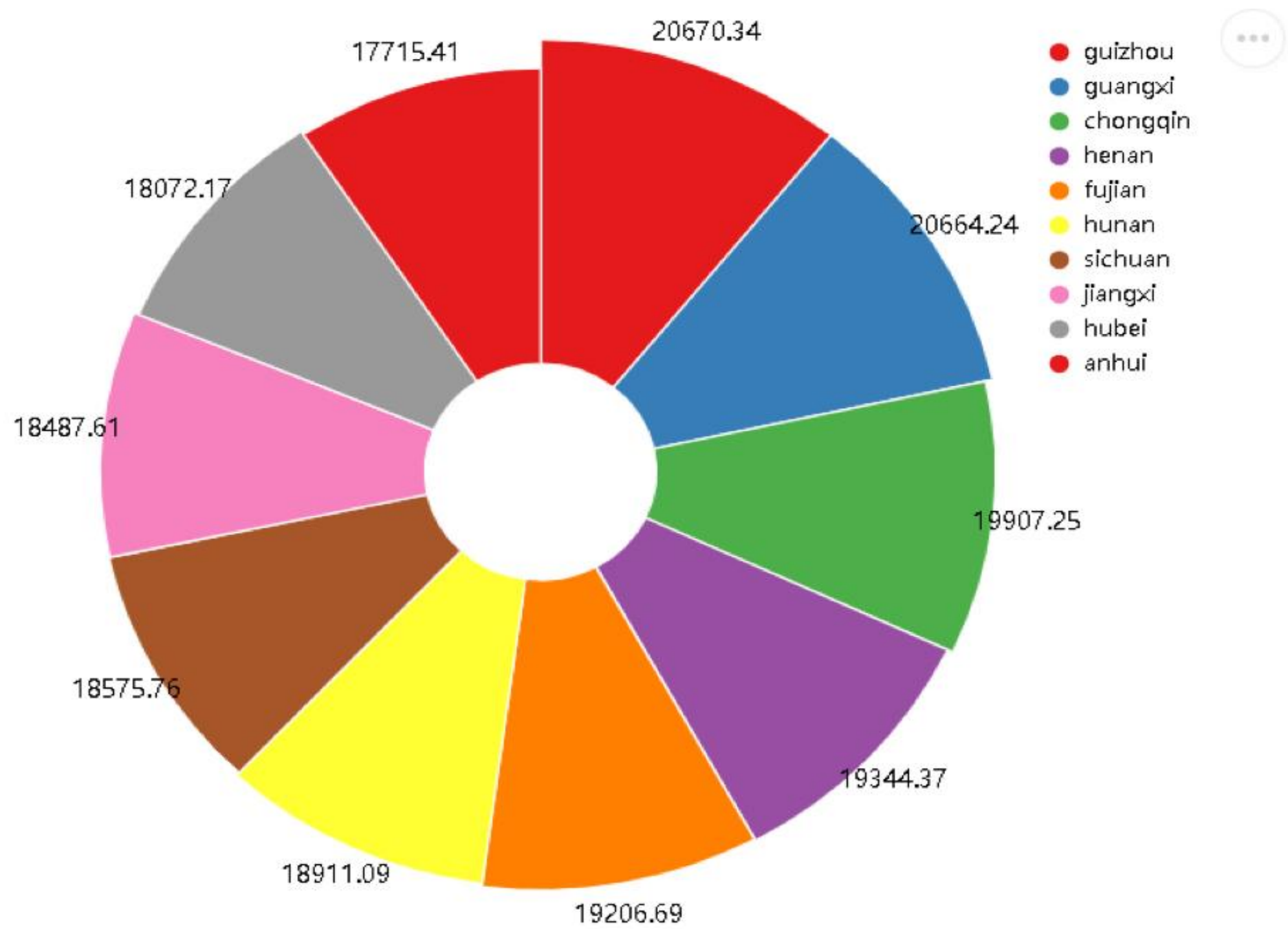

Note9. Analysis of Length of Stay Across Disease Categories Among Cross-Provincial Inpatients

disease categories H00-H59 and R00-R99 had the shortest average length of stay, while categories S00-T98 and U00-U99 had the longest average length of stay.(Table 5).

Table 5.Analysis of Hospitalization Days by Disease Spectrum Among Cross-Provincial Inpatients

ICD-10 Code	Systemic Disease	Avg. LOS（DAY)
A00-B99	Certain infectious and parasitic diseases	8.25
C00-D48	Neoplasms	8.76
D50-D89	Diseases of the blood and blood-forming organs and certain disorders involving the immune mechanism	7.36
E00-E90	Endocrine, nutritional, and metabolic diseases	8.14
F00-F99	Mental and behavioural disorders	5.96
G00-G99	Diseases of the nervous system	8.73
H00-H59	Diseases of the eye and adnexa	3.95
H60-H95	Diseases of the ear and mastoid process	6.97
I00-I99	Diseases of the circulatory system	8.32
J00-J99	Diseases of the respiratory system	7.78
K00-K93	Diseases of the digestive system	6.89
L00-L99	Diseases of the skin and subcutaneous tissue	8.47
M00-M99	Diseases of the musculoskeletal system and connective tissue	10.13
N00-N99	Diseases of the genitourinary system	7.08
O00-O99	Pregnancy, childbirth, and the puerperium	4.99
Q00-Q99	Congenital malformations, deformations, and chromosomal abnormalities	7.55
R00-R99	Symptoms, signs, and abnormal clinical and laboratory findings, not elsewhere classified	4.91
S00－T98	Injury, poisoning, and certain other consequences of external causes	12.76
U00-U99	Codes for special purposes	12.75
ZOO－Z99	Factors influencing health status and contact with health services	6.54
